# Supplementary material for: MetaRibo-Seq measures translation in microbiomes
Source: Nat Commun. 2020 Jun 29;11:3268. doi: 10.1038/s41467-020-17081-z (PMC7324362; doi:10.1038/s41467-020-17081-z)
Supplement: Supplementary file 10 — Supplementary Data 7 [file 41467_2020_17081_MOESM10_ESM.zip › File2/Confidence_High_Taxonomy/71221_out.krona.html]

Javascript must be enabled to view this page.

members
magnitude
magnitudeUnassigned
count
unassigned
taxon
rank

71221\_out

2

2
superkingdom
2

201174
2
phylum

class
2
1760

2
order
2037

family
2
2049

2
genus
1654

544580

SRS074682\_contig\_number\_contig-100\_17435.17435SRS147057\_contig\_number\_2151
species
2
